# Streamlined machine learning model for early sepsis risk prediction in burn patients

**DOI:** 10.1038/s41746-025-02078-z

**Published:** 2025-10-21

**Authors:** Marius Drysch, Felix Reinkemeier, Flemming Puscz, Jannik Hinzmann, German Burn Registry, Fuchs Paul Christian, Marcus Lehnhardt, Christoph Wallner, Sonja Verena Schmidt

**Affiliations:** 1 Department of Plastic Surgery, BG University Hospital Bergmannsheil, Ruhr University Bochum, Bochum, Germany; 2 Department of Plastic, Reconstructive, Hand and Burn Surgery, Hospital Cologne Merheim, University of Witten-Herdecke, Cologne, Germany

**Keywords:** Sepsis, Diseases, Health care, Medical research, Risk factors

## Abstract

Sepsis is the leading cause of mortality in burn patients, yet early identification remains difficult due to persistent hyperinflammatory responses and altered baseline physiology. We developed a streamlined machine learning model for early sepsis risk prediction in burn patients using data from 6629 patients across 11 centers participating in the German Burn Registry. The model was trained using only six admission-level variables (age, burned body surface area, deep partial-thickness burns, full-thickness burns, inhalation injury, and hypertension), selected through multiple feature selection methods and evaluated using cross-validated machine learning pipelines. The final Random Forest model achieved an AUROC of 0.91, sensitivity of 0.81, specificity of 0.85, and a negative predictive value of 0.98, enabling reliable early risk stratification immediately upon ICU admission. By relying solely on admission-level variables, this model offers a reliable and interpretable solution for early sepsis risk detection in burn patients, supporting timely interventions and potentially improving critical care outcomes.

## Introduction

Burn injuries are complex traumas that can vary widely in both severity and scope. Worldwide, up to 11 million burn injuries occur each year, resulting in ~180,000 burn-related deaths annually^1^. Sepsis and subsequent Multi-Organ Dysfunction Syndrome (MODS) remain the leading cause of mortality among adult burn patients, with reported death rates of up to 60%^2–4^. The early detection of sepsis, however, is particularly challenging in burn care which can mainly be attributed to two factors: First, extensive burns trigger a hypermetabolic response characterized by tachycardia, elevated temperature, and increased respiratory rate^5,6^. Consequently, burn patients with extensive injuries almost invariably meet SIRS criteria^6^, masking early signs of sepsis. Second, the loss of the primary protective barrier, the skin, exposes burn wounds to continuous microbial invasion as long as wounds remain open. Distinguishing between benign colonization and true invasive infection poses a major challenge, as colonization is common but can rapidly progress to infection if not promptly recognized^7^. Indeed, these complexities have motivated the development of separate, burn-specific consensus definitions such as those from the American Burn Association (ABA)^8^.

Despite tailored diagnostic guidelines, accurately predicting which patients are on a trajectory toward sepsis remains an unsolved clinical problem. Burn-specific scoring systems, such as the revised Baux and ABSI scores^9–12^ seek to predict mortality risk but do not account for the complexities of sepsis prediction in burn patients. While traditional scores are limited, machine learning (ML) has emerged as promising tool for advancing sepsis prediction by identifying subtle patterns of clinical deterioration in large datasets. For instance, He et al.^13^ developed an ensemble learning model that successfully predicted sepsis onset 6 h in advance in a general ICU population using electronic health records, achieving a sensitivity of 0.64 and specificity of 0.84. The unique pathophysiological changes in burn patients, however, complicate the application of standard ML models. Moreover, most existing models rely on extensive feature sets^14,15^ that are unavailable at admission, rendering them impractical for routine clinical use. Additionally, they frequently depend on dynamic data changes that manifest days to hours before sepsis onset^16,17^. Such limitations restrict the utility of these scores in providing actionable, early predictions.

Collectively, these challenges underscore the need for burn-specific predictive models. This study addresses these gaps by developing an ML model tailored to burn patients using data from the German Burn Registry. Given that the primary drivers of sepsis risk in burn patients, including injury severity and intrinsic patient factors, are established at admission, we hypothesized that a streamlined ML model could accurately stratify sepsis risk using only these admission-level variables.

## Results

### Baseline characteristics

The final patient cohort consisted of 6629 patients, with 521 (7.9%) developing sepsis during their hospital stay. Table 1 summarizes the baseline characteristics of the sepsis and non-sepsis groups. Patients in the sepsis group were generally older (mean ± SD: 55.0 ± 19.9 vs. 47.1 ± 19.3, *p* < 0.001) and had higher body mass indices (27.2 ± 5.7 vs. 26.4 ± 4.8, *p* < 0.001). Burn severity indicators, including burned body surface area (34.0% ± 21.4% vs. 10.4% ± 11.6%, *p* < 0.001) and deeper burns (e.g., burn depth 3: 16.1% ± 17.2% vs. 2.6% ± 7.4%, *p* < 0.001), were notably more common in sepsis patients. Inhalation injury and the need for ventilation were substantially more frequent in the sepsis group (45.5% vs. 11.6%, *p* < 0.001; 84.1% vs. 21.8%, *p* < 0.001, respectively). The average duration of ventilation was also significantly longer among sepsis patients (18.4 ± 21.3 days vs. 2.2 ± 6.0 days, *p* < 0.001). Patients in the non-sepsis group were more likely to have received cold water treatment (30.9% vs. 14.6%, *p* < 0.001). Accident context and cause of injury varied between groups. While flame burns were the most common cause in both groups, they occurred more frequently in sepsis patients (66.4% vs. 53.4%, *p* < 0.001). Injuries sustained during suicidal incidents were also more common in the sepsis group (9.8% vs. 3%, *p* < 0.001). Comorbidities such as diabetes mellitus (14.0% vs. 7.9%, *p* < 0.001), coronary heart disease (15.7% vs. 7.4%, *p* < 0.001), and hypertension (30.3% vs. 17.6%, *p* < 0.001) were more prevalent among sepsis patients. Additionally, kidney risk, defined as creatinine >1.5, was higher in the sepsis group (10.2% vs. 2.2%, *p* < 0.001). Pneumonia was more frequently diagnosed in sepsis patients (67.6% vs. 4.7%, *p* < 0.001), and mortality rates were significantly higher (38.0% vs. 4.3%, *p* < 0.001).

**Table 1:** Baseline characteristics of the study population

Feature	Non-sepsis	Sepsis	*p*-value
Number of patients	6108 (92.1%)	521 (7.9%)	
Male (%)	4281 (70.1%)	380 (72.9%)	0.188
Age (±SD)	47.1 ± 19.3	55.0 ± 19.9	<0.001
Height in m (±SD)	1.8 ± 0.1	1.8 ± 0.1	0.705
Weight in kg (±SD)	81.3 ± 17.0	83.8 ± 19.0	0.001
Body mass index (±SD)	26.4 ± 4.8	27.2 ± 5.7	<0.001
ABSI (±SD)	7.0 ± 2.6	10.8 ± 2.3	<0.001
Burn depth 2a (±SD)	4.2 ± 5.8	5.8 ± 10.3	<0.001
Burn depth 2b (±SD)	3.7 ± 5.9	12.2 ± 13.7	<0.001
Burn depth 3 (±SD)	2.6 ± 7.4	16.1 ± 17.2	<0.001
Burned body surface area (±SD)	10.4 ± 11.6	34.0 ± 21.4	<0.001
Accident context			
Home/leisure time (%)	4443 (72.7%)	347 (66.6%)	0.003
Work/school (%)	1182 (19.4%)	68 (13.1%)	<0.001
Suicidal (%)	183 (3.0%)	51 (9.8%)	<0.001
Criminal (%)	65 (1.1%)	16 (3.1%)	<0.001
Traffic (%)	47 (0.8%)	12 (2.3%)	<0.001
Cause of accident			
Flame (%)	3260 (53.4%)	346 (66.4%)	<0.001
Scalding (%)	1446 (23.7%)	70 (13.4%)	<0.001
Other (%)	56 (0.9%)	6 (1.2%)	0.766
Fat/Oil (%)	358 (5.9%)	7 (1.3%)	<0.001
Solid substance (Stove…) (%)	170 (2.8%)	14 (2.7%)	1
Liquid solid substance (%)	165 (2.7%)	7 (1.3%)	0.084
Acid (%)	104 (1.7%)	1 (0.2%)	0.014
Alkali (%)	56 (0.9%)	1 (0.2%)	0.141
Frostbite (%)	4 (0.1%)	0 (0.0%)	1
Pure IHT (%)	2 (0.0%)	0 (0.0%)	1
Risk factors			
Smoking (%)	1204 (19.7%)	119 (22.8%)	0.097
Diabetes mellitus (%)	484 (7.9%)	73 (14.0%)	<0.001
Peripheral arterial disease (%)	245 (4.0%)	41 (7.9%)	<0.001
Coronary heart disease (%)	453 (7.4%)	82 (15.7%)	<0.001
Hypertension (%)	1076 (17.6%)	158 (30.3%)	<0.001
COPD (%)	327 (5.4%)	46 (8.8%)	0.001
Arrhythmia (%)	280 (4.6%)	65 (12.5%)	<0.001
Heart failure (%)	250 (4.1%)	54 (10.4%)	<0.001
Obesity (%)	1015 (16.6%)	110 (21.1%)	0.01
Kidney risk (creatinine > 1.5) (%)	135 (2.2%)	53 (10.2%)	<0.001
Primary admission (%)	3986 (65.3%)	372 (71.4%)	0.005
Cold water treatment conducted (%)	1885 (30.9%)	76 (14.6%)	<0.001
Primary patients with cooling (%)	1262 (20.7%)	47 (9.0%)	<0.001
Ear temperature at admission ( ± SD)	36.5 ± 0.9	36.0 ± 1.2	<0.001
Inhalation injury (%)	711 (11.6%)	237 (45.5%)	<0.001
Bronchoscopy (%)	770 (12.6%)	247 (47.4%)	<0.001
IHT verified by bronchoscopy (%)	540 (8.8%)	215 (41.3%)	<0.001
Ventilated (%)	1331 (21.8%)	438 (84.1%)	<0.001
Number of days ventilated ( ± SD)	2.2 ± 6.0	18.4 ± 21.3	<0.001
Pneumonia (%)	290 (4.7%)	352 (67.6%)	<0.001
Deceased (%)	262 (4.3%)	198 (38.0%)	<0.001

Continuous variables are shown as mean ± SD, and categorical variables as counts (percentages).

### Feature selection

**Fig. 1:**
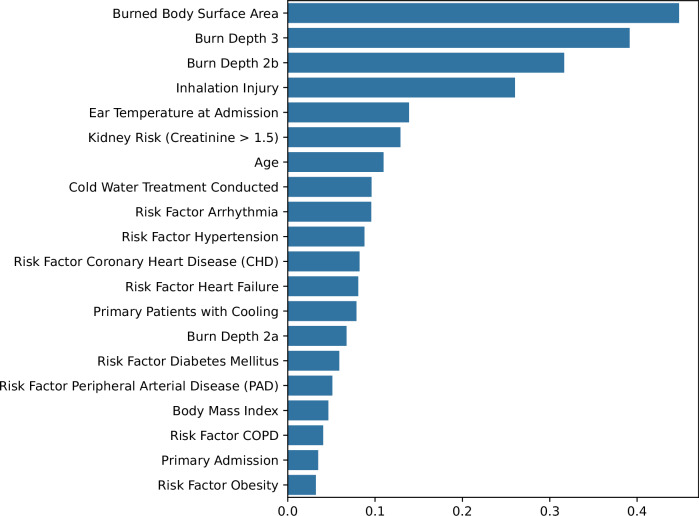
Pearson’s correlation coefficients (x-axis) for the top 20 clinical features (y-axis) associated with sepsis occurrence in burn patients. Burned body surface area, burn depth (full-thickness burns, burn depth 3; deep partial-thickness burns, burn depth 2b) and inhalation injury showed the strongest correlations, guiding feature selection for later ML model training.

Feature selection began with the identification of the top 20 features exhibiting the strongest correlation with sepsis, thereby establishing the foundation for subsequent refinement (Fig. 1). To ensure the development of robust and clinically relevant predictors, four selection methods (LASSO, ElasticNet, RFE, and RFECV) were employed, with each method optimized separately for AUC and recall. This process yielded eight distinct feature sets, and the consistency of feature selection across methods is summarized in Table 2. To evaluate the impact of feature selection on model performance and clinical feasibility, four feature sets were constructed. The *Intersection set* included the eight features selected by all automated methods: age, body mass index, deep partial thickness burns (burn depth 2b), full-thickness burns (burn depth 3), burned body surface area, cold water treatment conducted, ear temperature at admission, and inhalation injury. The *High Frequency set* comprised the 12 features selected by at least 75% of the methods. In addition to the features in the *Intersection set*, this included presence of kidney risk (creatinine > 1.5), arrhythmia, coronary heart disease, hypertension. The *Exploratory Data Analysis (EDA) set* was constructed to test a hypothesis-driven model based on six core clinical variables: deep partial thickness burns (burn depth 2b), full-thickness burns (burn depth 3), burned body surface area, inhalation injury, age, and hypertension. These features were selected based on their consistent identification as top predictors in our initial analysis and their established role in clinical burn assessment. Finally, the *Minimalistic set* was created by excluding inhalation injury and hypertension from the *EDA set* to test an even more streamlined model. Overall, these feature sets formed the basis for model development and evaluation.

**Table 2:** Frequency table of selected features

Feature	Lasso AUC	Lasso Recall	EN AUC	EN Recall	RFE AUC	RFE Recall	RCV AUC	RCV Recall	∑
Age	1	1	1	1	1	1	1	1	8
BD 2b	1	1	1	1	1	1	1	1	8
BD 3	1	1	1	1	1	1	1	1	8
BSA	1	1	1	1	1	1	1	1	8
CWTC	1	1	1	1	1	1	1	1	8
ET_Adm_	1	1	1	1	1	1	1	1	8
IHI	1	1	1	1	1	1	1	1	8
BMI	1	1	1	1	1	1	1	1	8
CHD	1	1	1	1	0	0	1	1	6
Kidney	1	1	1	1	0	0	1	1	6
HT	1	1	1	1	0	0	1	1	6
ARRHY	1	1	1	1	0	0	1	1	6
Obesity	1	1	1	1	0	0	1	0	5
HF	1	1	1	1	0	0	1	0	5
PA	0	1	0	1	1	1	1	0	5
Cooling	1	1	1	1	0	0	1	0	5
DM	0	1	1	1	0	0	1	0	4
BD 2a	0	0	0	0	1	1	1	1	4
COPD	0	1	0	1	0	0	1	0	3
PAD	0	1	1	1	0	0	0	0	3

*EN* ElasticNet, *RFE* Recursive Feature Elimination, *RCV* Recursive Feature Elimination with Cross-Validation, *AUC* Optimized for AUC, *Recall* Optimized for Recall, *BD 2b* Deep partial-thickness burns 2b, *BD 3* Full-thickness burns, *BSA* Burned Body Surface Area, *CWTC* Cold Water Treatment Conducted, *ET*_Adm_ Ear Temperature at Admission, *IHI* Inhalation Injury, *BMI* Body Mass Index, *CHD* Coronary Heart Disease, *Kidney* Kidney Risk (Creatinine >1.5), *HT* Risk Factor Hypertension, *ARRHY* Risk Factor Arrhythmia, *Obesity* Risk Factor Obesity, *HF* Risk Factor Heart Failure, *PA* Primary Admission, *Cooling* Primary Patients with Cooling, *Risk Factor DM* Diabetes Mellitus, *BD 2a* Burn Depth 2a, *COPD* Risk Factor COPD, *PAD* Risk Factor Peripheral Arterial Disease.

### Model performance

**Fig. 2:**
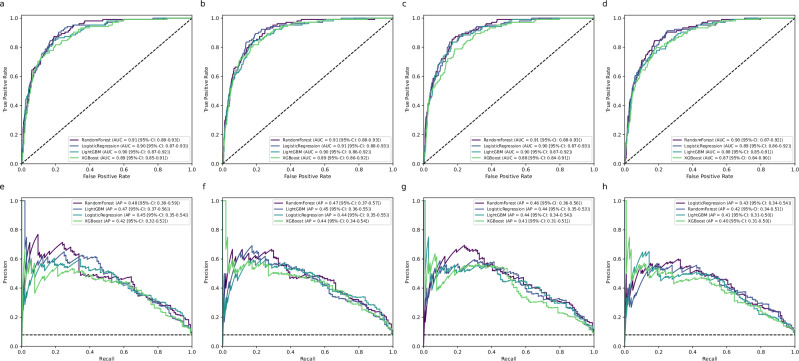
Performance comparison of machine learning models across four feature sets. Receiver Operating Characteristic (ROC) curves (top row; **a**–**d**) and Precision–Recall (PR) curves (bottom row; **e**–**h**) are shown for Random Forest, Logistic Regression, XGBoost, and LightGBM. Each column represents one feature set: *EDA* (**a**, **e**), *High Frequency* (**b**, **f**), *Intersection* (**c**, **g**), and *Minimalistic* (**d**, **h**). Legends indicate the area under the ROC curve (AUC) and the average precision (AP) with 95% confidence intervals. Random Forest and Logistic Regression consistently achieved the highest performance across all feature sets.

**Fig. 3:**
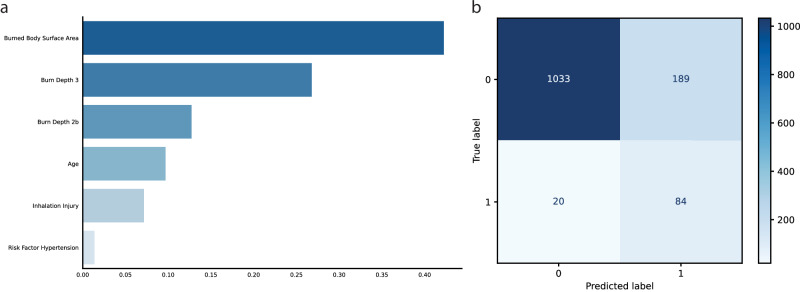
Feature importance and confusion matrix of the final Random Forest model trained with the *EDA* set predicting sepsis in burn patients. The left panel (**a**) presents feature importance scores, highlighting Burned Body Surface Area, full-thickness burns (Burn Depth 3), and deep partial-thickness burns (Burn Depth 2b) as the strongest predictors. The right panel (**b**) shows the confusion matrix, visualizing model accuracy, including true positive, true negative, false positive, and false negative predictions on the test dataset.

Performance of the four feature sets was evaluated using four machine learning algorithms, with results presented in Table 3 and additional metrics in Fig. 2 (for further detailed information see Supplementary Table 2). The *EDA set*, comprising six core clinical features, demonstrated the strongest performance, with Random Forest achieving an AUROC of 0.91, sensitivity of 0.81 and specificity of 0.85. Logistic Regression performed similarly, with an AUROC of 0.90, sensitivity of 0.81 and specificity of 0.85. The *High Frequency set*, with 12 features, achieved comparable metrics (Logistic Regression: AUROC 0.91, sensitivity 0.84, specificity 0.85; Random Forest: AUROC 0.91, sensitivity 0.80, specificity 0.85) but offered limited incremental benefit over the EDA set. While Random Forest and Logistic Regression performed robustly, the addition of more features did not significantly improve predictive accuracy, suggesting diminishing returns beyond a core subset of variables. The *Intersection set*, containing 8 features, also showed high predictive value, with Random Forest achieving an AUROC of 0.91, sensitivity of 0.77 and specificity of 0.86, slightly below the EDA set. The *Minimalistic set*, containing only four features, maintained competitive performance, with Random Forest achieving an AUROC of 0.90, sensitivity of 0.78 and specificity of 0.84. While slightly less sensitive than the richer feature sets, its simplicity and comparable AUROC make it an attractive alternative in resource-limited settings or for rapid integration into workflows. Across all sets, Random Forest and Logistic Regression consistently delivered high sensitivity (up to 0.84) and strong AUROC values (up to 0.91). This emphasis on sensitivity resulted in slightly lower PPV (often around 0.32–0.35) but ensured very high NPV (up to 0.98), underscoring their strength in identifying patients at low risk for sepsis. Overall, the *EDA set*, trained with Random Forest (see Fig. 3), emerged as the optimal choice, balancing predictive accuracy and clinical practicality. This model achieved an AUROC of 0.91, sensitivity of 0.81, specificity of 0.85 and NPV of 0.987, reinforcing its potential for early sepsis prediction in clinical settings.

**Table 3:** Performance metrics of machine learning models across feature sets

Set	Model	Accuracy	Sensitivity	Specificity	PPV	NPV	AUC
EDA (6 features)	LogisticRegression	0.848	0.808	0.852	0.317	0.981	0.901
	RandomForest	0.842	0.808	0.845	0.308	0.981	0.908
	LightGBM	0.849	0.769	0.856	0.313	0.978	0.898
	XGBoost	0.854	0.692	0.867	0.308	0.971	0.876
HighFrequency (12 features)	LogisticRegression	0.851	0.837	0.853	0.326	0.984	0.907
	RandomForest	0.847	0.798	0.851	0.313	0.980	0.908
	XGBoost	0.870	0.731	0.882	0.345	0.975	0.895
	LightGBM	0.862	0.712	0.875	0.326	0.973	0.896
Intersection (8 features)	LogisticRegression	0.854	0.788	0.859	0.323	0.979	0.905
	RandomForest	0.852	0.769	0.859	0.317	0.978	0.908
	XGBoost	0.864	0.740	0.875	0.335	0.975	0.886
	LightGBM	0.870	0.740	0.881	0.345	0.976	0.896
Minimalistic (4 features)	RandomForest	0.838	0.779	0.843	0.297	0.978	0.898
	LightGBM	0.839	0.740	0.847	0.292	0.975	0.880
	LogisticRegression	0.842	0.721	0.853	0.294	0.973	0.892
	XGBoost	0.845	0.712	0.857	0.297	0.972	0.873

### Explainability and further insights

**Fig. 4:**
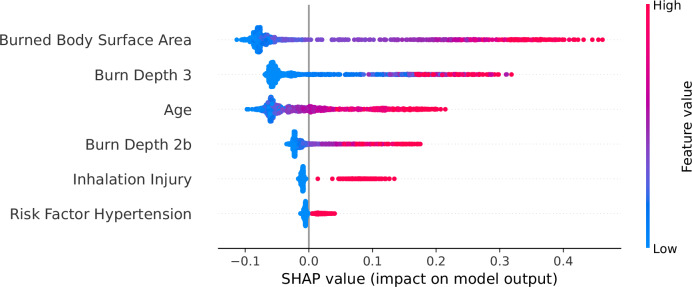
SHAP summary plot demonstrating feature influence on the Random Forest model’s predictions for sepsis in burn patients. Individual dots represent patient-level data, colored by the value of each feature, with red indicating higher values and blue indicating lower values. Features ranked by overall importance reveal Burned Body Surface Area, Full-thickness Burns (Burn Depth 3), and Age as most important for sepsis prediction.

**Fig. 5:**
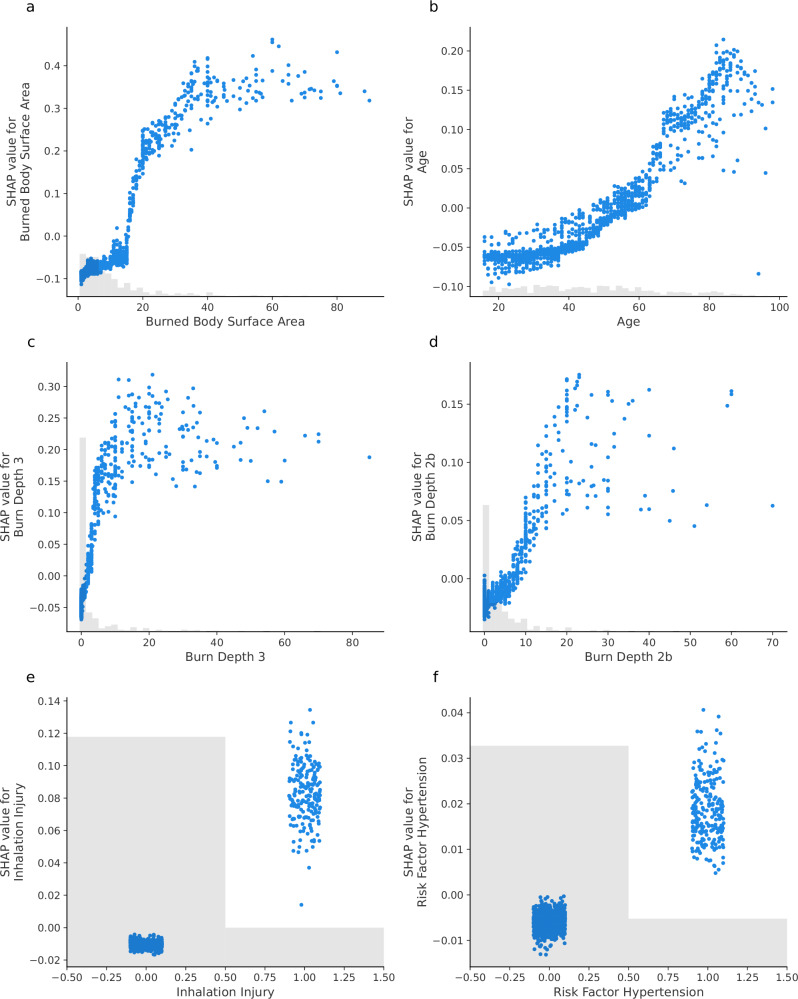
Partial dependence plots illustrating the influence of individual features on sepsis prediction. Each plot shows how the value of a single feature (x-axis) impacts its contribution to the model’s output (y-axis, SHAP value) for every patient. Higher SHAP values push the prediction towards a higher risk of sepsis. The features from the final Random Forest model are: **a** Burned Body Surface Area, **b** Age, **c** full-thickness burns (Burn Depth 3), **d** deep partial-thickness burns (Burn Depth 2b), **e** Inhalation Injury, and **f** Hypertension.

SHAP was employed to analyze feature contributions and interactions within the predictive model. Globally, the SHAP summary dot plot (Fig. 4) reveals that burned body surface area, full-thickness burns and age are the most impactful features driving model predictions, followed by deep partial-thickness burns, inhalation injury and hypertension. Burned body surface area consistently emerges as the most dominant predictor, with positive SHAP values indicating its strong association with increased sepsis risk. Age and full-thickness burns demonstrate a gradient effect, where higher values substantially contribute to sepsis risk, highlighting their clinical relevance. Deep partial-thickness burns, inhalation injury and hypertension provide more nuanced contributions, reflecting the multifaceted nature of sepsis risk factors in burn patients. These global patterns are supported by the SHAP summary bar plot, which ranks features by their average contribution to the model and is included in the Supplementary Information (Supplementary Fig. 1). Partial dependence plots (Fig. 5) reveal critical insights into the relationships between individual features and the predicted risk of sepsis. These findings align with clinical expectations and provide actionable interpretations of the model’s behavior. Burned body surface area demonstrates a sharp increase at around 20–30% and displays a clear ceiling effect. The SHAP values increase steeply up to ~40–50% total body surface area but plateau thereafter. For full-thickness burns, the predictive contributions rise sharply even with minimal increases in severity. By contrast, deep partial-thickness burns exhibit a more gradual relationship, with smaller contributions to risk that align with the less severe physiological impact of partial-thickness burns. Age demonstrates a nonlinear risk pattern, with minimal impact on model predictions in younger patients, followed by a progressively increasing contribution beginning around age 40. Binary features such as inhalation injury and hypertension exhibit distinct step-like changes in SHAP values, suggesting strong categorical impacts on predictions. However, dependence plots (Supplementary Figs. 2 and 3), employed to further elucidate potential nonlinear influences from other features, did not show conclusive results regarding these interactions.

## Discussion

Sepsis is the leading cause of death in burn patients, yet early detection remains a major challenge. In our dataset, sepsis was associated with a mortality rate of 38%, underscoring the need for burn-specific sepsis prediction models. Our machine learning-based model, tailored to this population, achieves high accuracy (AUROC 0.91, Sensitivity 0.81, Specificity 0.85) while relying only on six admission-level features, making it immediately applicable for early risk stratification. Despite its streamlined design, the model demonstrates strong diagnostic performance, particularly in ruling out sepsis (NPV 0.98), reinforcing its potential as a highly reliable screening tool.

Existing sepsis prediction models vary widely in feature selection, data dependencies, and performance. Most achieve AUROC values between 0.40 and 0.98 but often rely on post-admission data, real-time vital signs, or evolving laboratory trends, limiting their applicability at admission. While effective in general ICU settings, these models typically have not been validated in burn patients. Kim et al.^18^ used 40 features in their final model, achieving high accuracy in predicting Sepsis 3 h prior to onset (AUROC 0.94, Sensitivity 0.93, Specificity 0.91) but requiring continuous monitoring. Similarly, Shashikumar et al.^19^ leveraged post-admission data with a total number of 40 features, improving predictions for a 12 h lead time (AUROC 0.95, Sensitivity 91.6, Specificity 93.0) but, again, restricting usability. Feature efficiency improves real-time applicability but may come at the expense of predictive strength. Cesario et al.^20^ demonstrated that 16 well-chosen features yield high specificity but reduced sensitivity (AUROC 0.97, Sensitivity 0.61, Specificity 0.99), while Al-Mualemi et al.^21^, using only 7 features, showed a drop in predictive strength (AUROC 0.78, Sensitivity 0.93, Specificity 0.93). Balancing sensitivity and specificity is another key consideration. High-specificity models often sacrifice sensitivity, delaying sepsis detection, whereas high-sensitivity models may generate more alerts, leading to an increased need for clinical interpretation. Giannini et al.^22^ illustrate how prioritizing specificity may risk missing cases (AUROC 0.88, Sensitivity 0.26, Specificity 0.98), whereas Yang et al.^14^ demonstrates the opposite trade-off, achieving high sensitivity at the expense of specificity (AUROC 0.85, Sensitivity 0.90, Specificity 0.64). Our model, while streamlined, achieves AUROC comparable to high-dimensional models without relying on ICU-dependent variables, striking a balanced approach that supports early identification of at-risk patients.

The SHAP analysis provides an opportunity to explore the model’s decision-making process, revealing patterns that align with, yet also appear to refine, established clinical knowledge. For instance, while it is well-established that a large TBSA is a major risk factor for sepsis^6,23^, the model indicates a potential ceiling effect, where the predictive influence of TBSA plateaus in very extensive burns (40–50%). This observation may suggest that for this specific subgroup, other variables beyond burn size become the dominant drivers of risk. Consistent with prior evidence^2^, the model assigns a high predictive value to full-thickness burns, illustrating a steep contribution curve such that even small increases significantly elevate the predicted sepsis risk. The model also identifies a nonlinear relationship with age, with risk accelerating more sharply after 40 years of age. This ability to model complex, nonlinear relationships, which are often challenging to capture with traditional scoring systems, may represent a key advantage of the present approach.

It is crucial to emphasize that this model is designed as a risk stratification tool based on static admission characteristics, not as a dynamic, real-time diagnostic tool. A central clinical challenge in burn care is distinguishing the persistent hypermetabolic state from the acute onset of sepsis. The model does not address this specific diagnostic dilemma at the moment sepsis is suspected. Instead, its purpose is to identify, immediately upon admission, which patients are at high risk of developing sepsis at any point during their hospital stay. Understanding the clinical meaning of this risk stratification requires interpreting the model’s performance metrics. While the model’s PPV is modest at 0.31, this is a known challenge in predicting low-prevalence events and is consistent with findings from comparable models. Shashikumar et al., for instance, reported PPVs of 0.38 and 0.20 for their sepsis prediction algorithm^19^. Additionally, a post-hoc analysis of the predictions made by the Random Forest model revealed that patients identified as high risk who did not develop sepsis (false positives) had a mortality rate of 20.6% which is significantly lower than that of the true positive cohort (39.3%, Supplementary Fig. 4). These findings suggest the model identifies a clinically relevant intermediate-risk population with severe baseline characteristics, despite not meeting formal sepsis criteria during their ICU stay. However, a high-risk alert should still not be an indication for immediate therapeutic intervention. Initiating antimicrobial therapy for instance, based solely on the model’s flag, would be premature given the modest PPV and would risk significant antibiotic overuse. Rather, it should trigger a state of heightened surveillance. For the high-risk cohort, a lower threshold for specific diagnostic tests and implementing advanced monitoring may be a more reasonable approach. This could include integrating more frequent assessment of inflammatory biomarkers such as leukocyte count, C-reactive protein and procalcitonin as well as incorporation of emerging sepsis markers, e.g., interleukin-6 (IL-6)^24^, presepsin^25^ or pancreatic stone protein (PSP)^26^. It could also mean enhancing microbiologic surveillance, e.g., with early blood cultures or tracheal sampling. Finally, the model may help individualize the decision to initiate advanced hemodynamic monitoring (e.g., transpulmonary thermodilution). While some centers use thresholds such as a TBSA of 20%^27^, indications for advanced monitoring vary and the decision often requires clinical judgment. In these borderline cases our model could provide data-driven support to escalate monitoring, thereby facilitating targeted fluid resuscitation and closer metabolic surveillance. In contrast, the high NPV (>0.98) provides a more direct and arguably more robust rationale for clinical action, specifically for evidence-based restraint. The high degree of certainty may support the clinical team in adhering to antibiotic stewardship principles. In the common scenario of a burn patient exhibiting ambiguous inflammatory signs, the model provides objective data that can reinforce a decision to continue observation rather than initiating antimicrobial therapy. While the model should never override acute clinical judgment, it can be a valuable tool in navigating these frequent diagnostic gray zones.

Despite its strengths, this study also faces limitations. A key limitation is the lack of external validation, as the model’s performance has been assessed only within this cohort. Its generalizability to other populations, healthcare settings, and regions remains untested. Another limitation is the lack of a universally accepted sepsis definition for burn patients. The Sepsis-3 criteria were developed for general ICU populations while clinical and pathophysiological differences in burn patients may influence classification^28–30^. In this study, sepsis definition was based on the ABA consensus criteria^8^, which includes criteria such as signs of hypermetabolism (i.e., progressive tachycardia, progressive tachypnea), metabolic derangements (i.e., hyperglycemia) and a required documented infection. While it is commonly used in burn care, the model’s performance may differ if applied in centers that utilize alternative diagnostic criteria for sepsis.

The next step is to make the model publicly accessible to facilitate broader validation and integration into clinical practice. Plans are underway to host the model and its documentation on our institution’s official website, enabling healthcare providers and researchers worldwide to access and utilize it. By fostering transparency and collaboration, the study aims to refine the model further through external validation and user feedback, ultimately enhancing its utility in diverse clinical settings.

In summary, this study introduces a tailored machine learning model for early sepsis prediction in burn patients, achieving high accuracy (AUROC 0.91) using only six admission-level features. By enabling real-time risk stratification without relying on post-admission data, the model offers a practically applicable and interpretable tool for early risk stratification. While external validation is needed, its strong performance and ease of integration highlight its potential to enhance sepsis detection and improve patient outcomes in burn ICUs.

## Methods

### Data source and study design

The present study employed a retrospective cohort design, based on data extracted from the German Burn Registry (GBR). The GBR, a national database maintained by the German Society for Burn Medicine (DGV), systematically collects data on patients admitted to intensive care units in specialized burn centers across Germany. For the present study, data spanning the period from January 2015 to December 2023 were analyzed, representing 11 participating burn centers for adults.

The GBR database contains over 60 variables for each patient, including demographic characteristics, burn-related details (e.g., burned body surface area [TBSA], burn depth), physiological parameters, treatment interventions, and clinical outcomes. Data collection is performed prospectively by trained personnel at each center using a standardized documentation protocol. The infrastructure for data capture and management is provided by the VR-DGV Committee, using an online randomization tool (MOSAIC) integrated with an open-source clinical trial management platform (OpenClinica^31^). Scientific data analysis was approved according to a peer review procedure following the rules of the publication guideline of the VR-DGV (VR-DGV-project-ID: 2024-02). All patients included in this study received care in accordance with national guidelines and established clinical pathways. A brief review of the current standard of care in German burn centers, including typical fluid resuscitation, debridement practices, and the common infection sites associated with sepsis in this population, is provided in the Supplementary Information.

### Data preprocessing

The initial dataset comprised 10,067 patients. Inclusion criteria stipulated that patients must have complete records for sepsis status and sufficient data on key clinical variables. Patients with missing sepsis outcome, those with blistering skin diseases and those who died within 24 hours of admission were excluded. After applying these criteria, 6629 patients were retained for analysis. Missing values were handled based on variable type and expert knowledge. Variables critical for the analysis were imputed using clinically appropriate methods, such as mean imputation for continuous data and mode imputation for categorical data (for a detailed summary see Supplementary Table 1). Features that were deemed irrelevant or nonsensical for predicting sepsis, such as the day of the week, were excluded when building the ML model, along with those associated with post-admission outcomes or at risk of data leakage (e.g., ventilated, pneumonia, deceased). Sepsis, the primary outcome, was defined as the occurrence of sepsis at any point during the patient’s initial hospital stay. The diagnosis, as documented in the registry, is based on the 2007 American Burn Association (ABA) consensus criteria^8^ for sepsis and infection in burns. The dataset was split into training (80%) and test (20%) sets using stratified sampling to preserve the distribution of outcomes. All preprocessing was performed on anonymized data in compliance with national data protection regulations.

### Machine learning workflow

For feature selection, the top 20 features most strongly correlated with the outcome variable were identified, forming the basis for further refinement. Four feature sets were constructed based on their selection frequency across methods, including LASSO regression, ElasticNet, recursive feature elimination (RFE), and recursive feature elimination with cross-validation (RFECV)^32–35^. Each feature set was used to train four machine learning algorithms: Random Forest, Logistic Regression, XGBoost, and LightGBM^36–39^.

Model training was conducted using the training dataset (80%) with five-fold stratified cross-validation to ensure thorough evaluation and prevent overfitting. Particular emphasis was placed on recall during both training and hyperparameter optimization, as early detection of the outcome (sepsis) required minimizing false negatives. Hyperparameter optimization was performed using GridSearchCV. The performance of each model was assessed on the test dataset (20%) using metrics including the area under the receiver operating characteristic curve (AUROC), precision–recall area under the curve (PR-AUC), sensitivity, specificity, positive predictive value (PPV), negative predictive value (NPV), F1-score, and F2-score.

To enhance interpretability of the models, SHAP (SHapley Additive exPlanations)^40^ was employed to quantify the contribution of individual features to the predictions. Global feature importance was visualized using bar and dot plots, while local predictions were illustrated through waterfall plots. Dependence plots and partial dependence plots were used to explore feature interactions, providing deeper insights into how specific predictors influenced model outputs.

**Fig. 6:**
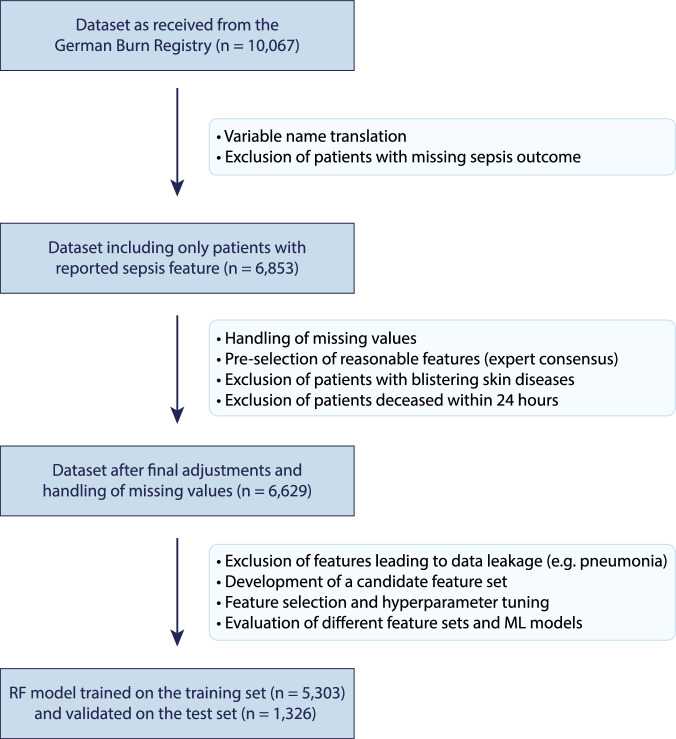
Flowchart of patient selection, data preprocessing, and model training. From 10,067 patients initially retrieved from the German Burn Registry, patients without documented sepsis status, those with blistering skin disease and those deceased within 24 h were excluded. After further preprocessing, the final dataset (*n* = 6629) underwent feature selection and machine learning model development. The Random Forest model was trained on 5303 patients and validated on 1326 patients.

Receiver operating characteristic and precision–recall curves were generated for all models and feature sets, with bootstrapped 95% confidence intervals. Confusion matrices were used to illustrate classification performance. The final model was selected based on its performance metrics, interpretability, and clinical feasibility, ensuring both predictive accuracy and practical applicability in critical care settings. The overall workflow can be found in Fig. 6.

### Statistical analysis

Continuous variables were summarized as means with standard deviations (SD), while binary and categorical variables were presented as absolute counts and frequencies (percentages). Comparisons between the sepsis and non-sepsis group were performed based on the type and distribution of variables. For continuous variables, Student’s *t* test was used to compare means when data were normally distributed, while the Mann–Whitney U test was applied for non-normally distributed data. Normality of the data was assessed using the Shapiro–Wilk test, and the equality of variances was evaluated using Levene’s test. For binary and categorical variables, the Chi-square test was used to assess group differences when expected cell frequencies were sufficient. For contingency tables with low expected frequencies, Fisher’s exact test was applied. All statistical tests were two-sided, and a *p*-value of <0.05 was considered statistically significant. The analyses were conducted using Python 3.9 and respective libraries.

### Ethics approval and consent to participate

This study was conducted in accordance with the ethical principles outlined in the Declaration of Helsinki. Participating patients provided general consent for their data to be recorded and used for scientific purposes. The study protocol was reviewed and approved by the Ethics Committee of Ruhr University Bochum (Reference Number: 21-7184). As this was a retrospective study using anonymized registry data, additional informed consent was not required.

## Supplementary information

Supplementary information

## Data Availability

The dataset analyzed in this study was obtained from the GBR and is not publicly available due to patient confidentiality and data protection regulations. Researchers interested in accessing the dataset may submit a formal request to the DGV in accordance with the registry’s data-sharing policies. Further details regarding data access and usage conditions can be found on the official DGV website (https://verbrennungsmedizin.de).

## Code availability

The custom code used for all stages of data analysis in this study, including data preprocessing, feature selection, model training, hyperparameter tuning, and generation of figures, is available from the corresponding author upon reasonable request. The analysis was conducted using Python (version 3.9). The machine learning pipeline was built primarily using the scikit-learn library for implementing Logistic Regression, Random Forest, RFE, RFECV, and GridSearchCV. Other key libraries include XGBoost, LightGBM, SHAP (for model interpretability and explainability plots), pandas (for data manipulation), and matplotlib/seaborn (for data visualization). Specific library versions can be provided along with the code.
