# Supplementary material for: Streamlined machine learning model for early sepsis risk prediction in burn patients
Source: NPJ Digit Med. 2025 Oct 21;8:621. doi: 10.1038/s41746-025-02078-z (PMC12540978; doi:10.1038/s41746-025-02078-z)
Supplement: Supplementary file 1 — Supplementary information [file 41746_2025_2078_MOESM1_ESM.pdf]

Supplementary Information for "Streamlined Machine Learning Model for Early Sepsis Risk Prediction in Burn Patients"

Marius Drysch[1], Felix Reinkemeier[1], Flemming Puscz[1], Jannik Hinzmann[1], German Burn Registry[#], Marcus Lehnhardt[1], Christoph Wallner[1†], Sonja Verena Schmidt[1†]*

[1] Department of Plastic Surgery, BG University Hospital Bergmannsheil, Ruhr University Bochum, 44789 Bochum, Germany

[#] A list of authors and their affiliations appears at the end of the main publication.

[*] corresponding author

† both authors contributed equally

Correspondence: marius.drysch@bergmannsheil.de

Keywords: Burns, sepsis, prediction, risk stratification, machine learning

Feature	Missing Values	Percentage (%)	Impute strategy
Total Human Albumin Administration (20%) within the First 24 Hours Post-Accident	6300	91.93	Exclude feature
Total Human Albumin Administration (20%) within the First 48 Hours Post-Accident	6096	88.95	Exclude feature
Length of Stay on burn ICU (Old)	5523	80.59	Exclude feature
Blood Alcohol Content	4539	66.23	Impute Mode
Total Crystalloid Administration within the First 48 Hours Post-Accident	4412	64.38	Exclude feature
Total Crystalloid Administration within the First 24 Hours Post-Accident	4232	61.75	Exclude feature
Kidney Risk (Creatinine > 1.5)	3646	53.20	Impute Mode
Preclinical Volume of Crystalloids/Colloids Administered	3193	46.59	Exclude feature
Primary Patients with Cooling 0/1	2489	36.32	Impute Mode
Deceased within 24 hours	2039	29.75	Exclude feature
Deceased within a Week	2031	29.64	Exclude feature
Catecholamine Administration within the First 24 Hours Post-Accident (Yes Only for Continuous Administration of Catecholamines >1h)	1598	23.32	Exclude feature
Total Number of Surgeries Including Admission Bath Until Transfer from SV Station	1366	19.93	Exclude feature
Ear Temperature at Admission	1345	19.63	Impute Mean
Risk Factor Coronary Heart Disease (CHD)	1343	19.60	Impute Mode
Risk Factor Smoking	1343	19.60	Impute Mode
Risk Factor Diabetes Mellitus	1343	19.60	Impute Mode
Risk Factor Peripheral Arterial Disease (PAD)	1343	19.60	Impute Mode
Risk Factor Heart Failure	1343	19.60	Impute Mode
Risk Factor Arrhythmia	1343	19.60	Impute Mode
Risk Factor COPD	1343	19.60	Impute Mode
Risk Factor Hypertension	1343	19.60	Impute Mode
Number of Days Ventilated (Including Partial Days)	1332	19.44	Impute Mean
Cold Water Treatment Conducted	1042	15.21	Impute Mode
Risk Factor Obesity	554	8.08	Impute Mode
Body Mass Index	554	8.08	Impute Mean
Height in m	522	7.62	Impute Mean

Weight in kg	496	7.24	Impute Mean
Cause of Accident_New	417	6.08	Impute 15 = "unknown"
Cause = Electricity	417	6.08	Exclude feature
Admission Hour	101	1.47	Impute Mean
Referral	100	1.46	Impute Mode
Primary Admission	66	0.96	If referral = 1, impute 1, otherwise 0
ABSI: Age Points	22	0.32	Set to age divided by 10
ABSI Recalculated	22	0.32	Sum of the other ABSI features in that row
Age in Groups	22	0.32	Impute mean
Age (Completed)	22	0.32	Impute mean
Length of Stay in Hours	6	0.09	Impute mean
Pneumonia	2	0.03	Impute mode

Supplementary Table 1. Frequency of Missing Features and Imputation Strategies. Table detailing the percentage of missing values for each variable in the dataset containing patients with a reported sepsis feature (n=6,853) and the corresponding strategy used for handling the missing data (e.g., mean/mode imputation, exclusion).

Subset	Classifier	#	Features	BestParams	Accuracy	Sensitivity	Specificity	PPV	NPV	F1_1	F1_0	F2_1	F2_0	AUC
EDA	LR	6	['Burned Body Surface Area', 'Burn Depth 3', 'Burn Depth 2b', 'Inhalation Injury', 'Age', 'Risk Factor Hypertension']	{'C': 1.0, 'class_weight': 'balanced'}	0.848	0.808	0.852	0.317	0.981	0.455	0.912	0.617	0.875	0.901
EDA	RF			{'class_weight': 'balanced', 'max_depth': 5, 'n_estimators': 200}	0.842	0.808	0.845	0.308	0.981	0.446	0.908	0.610	0.869	0.908
EDA	LGBM			{'class_weight': 'balanced', 'max_depth': 5, 'n_estimators': 100}	0.849	0.769	0.856	0.313	0.978	0.444	0.913	0.595	0.878	0.898
EDA	XGB			{'max_depth': 3, 'n_estimators': 100, 'scale_pos_weight': 10}	0.854	0.692	0.867	0.308	0.971	0.426	0.916	0.554	0.886	0.876
HighFreq	LR	12	['Age', 'Body Mass Index', 'Burn Depth 2b', 'Burn Depth 3', 'Burned Body Surface Area', 'Cold Water Treatment Conducted', 'Ear Temperature at Admission', 'Inhalation Injury', 'Kidney Risk (Creatinine > 1.5)', 'Risk Factor Arrhythmia', 'Risk Factor Coronary Heart Disease (CHD)', 'Risk Factor Hypertension']	{'C': 1.0, 'class_weight': 'balanced'}	0.851	0.837	0.853	0.326	0.984	0.469	0.914	0.637	0.876	0.907
HighFreq	RF			{'class_weight': 'balanced', 'max_depth': 5, 'n_estimators': 200}	0.847	0.798	0.851	0.313	0.980	0.450	0.911	0.609	0.874	0.908
HighFreq	XGB			{'max_depth': 3, 'n_estimators': 100, 'scale_pos_weight': 10}	0.870	0.731	0.882	0.345	0.975	0.469	0.926	0.597	0.899	0.895
HighFreq	LGBM			{'class_weight': 'balanced', 'max_depth': 5, 'n_estimators': 100}	0.862	0.712	0.875	0.326	0.973	0.447	0.921	0.575	0.893	0.896

Intersection	LR	8	['Age', 'Body Mass Index', 'Burn Depth 2b', 'Burn Depth 3', 'Burned Body Surface Area', 'Cold Water Treatment Conducted', 'Ear Temperature at Admission', 'Inhalation Injury']	{'C': 0.1, 'class_weight': 'balanced'}	0.854	0.788	0.859	0.323	0.979	0.458	0.915	0.612	0.881	0.905
Intersection	RF			{'class_weight': 'balanced', 'max_depth': 5, 'n_estimators': 200}	0.852	0.769	0.859	0.317	0.978	0.449	0.915	0.599	0.881	0.908
Intersection	XGB			{'max_depth': 3, 'n_estimators': 100, 'scale_pos_weight': 10}	0.864	0.740	0.875	0.335	0.975	0.461	0.922	0.596	0.893	0.886
Intersection	LGBM			{'class_weight': 'balanced', 'max_depth': 5, 'n_estimators': 100}	0.870	0.740	0.881	0.345	0.976	0.471	0.926	0.603	0.898	0.896
Minimalistic	RF	4	['Age', 'Burn Depth 2b', 'Burn Depth 3', 'Burned Body Surface Area']	{'class_weight': 'balanced', 'max_depth': 5, 'n_estimators': 200}	0.838	0.779	0.843	0.297	0.978	0.430	0.905	0.588	0.867	0.898
Minimalistic	LGBM			{'class_weight': 'balanced', 'max_depth': 5, 'n_estimators': 100}	0.839	0.740	0.847	0.292	0.975	0.418	0.906	0.566	0.870	0.880
Minimalistic	LR			{'C': 0.01, 'class_weight': 'balanced'}	0.842	0.721	0.853	0.294	0.973	0.418	0.909	0.559	0.874	0.892
Minimalistic	XGB			{'max_depth': 3, 'n_estimators': 100, 'scale_pos_weight': 10}	0.845	0.712	0.857	0.297	0.972	0.419	0.911	0.556	0.878	0.873

Supplementary Table 2. **Comprehensive Performance Data for All Trained Models.** Table detailing the performance of all four machine learning algorithms (Random Forest, Logistic Regression, XGBoost, LightGBM) across all four feature sets (EDA, High Frequency, Intersection, Minimalistic).

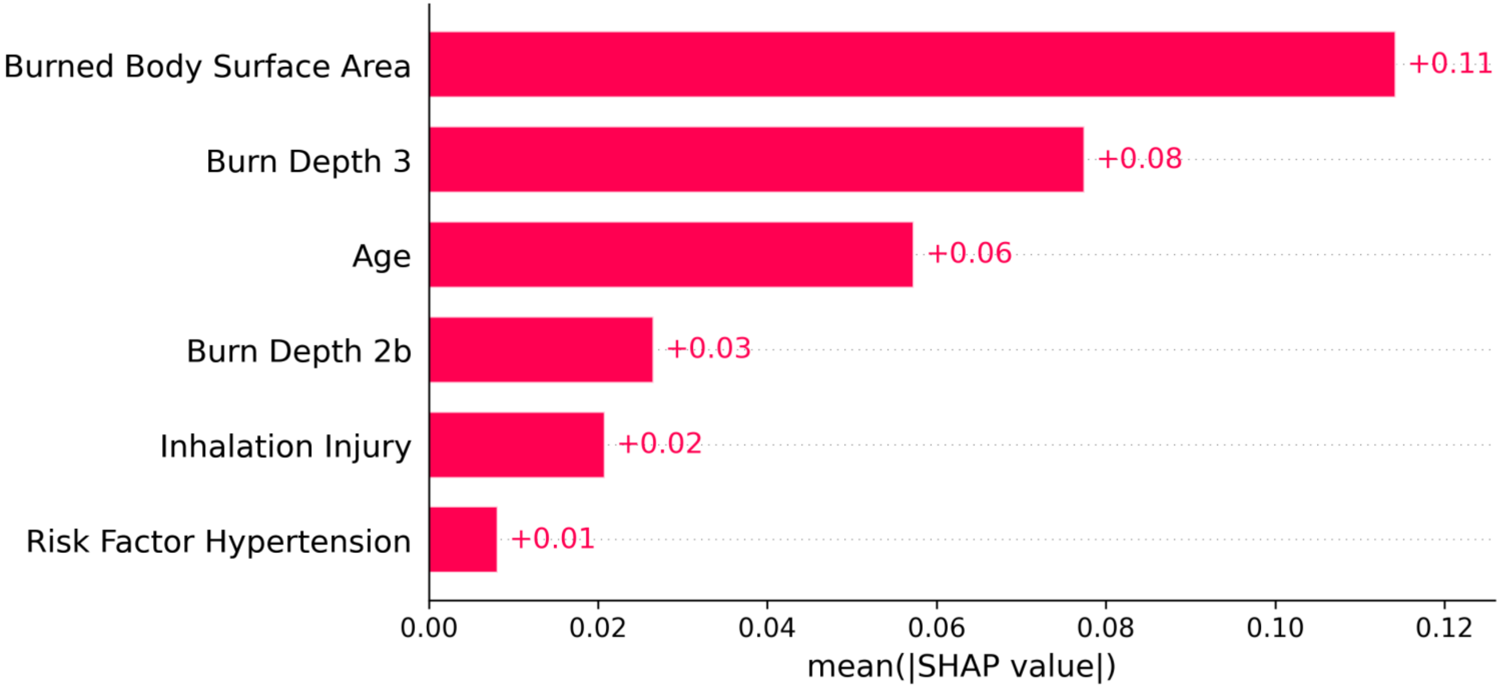

Supplementary Figure 1. Global Feature Importance Based on SHAP Values. Bar chart ranking the six features of the final Random Forest model by their mean absolute SHAP value, representing a feature's average impact on the model's output magnitude.

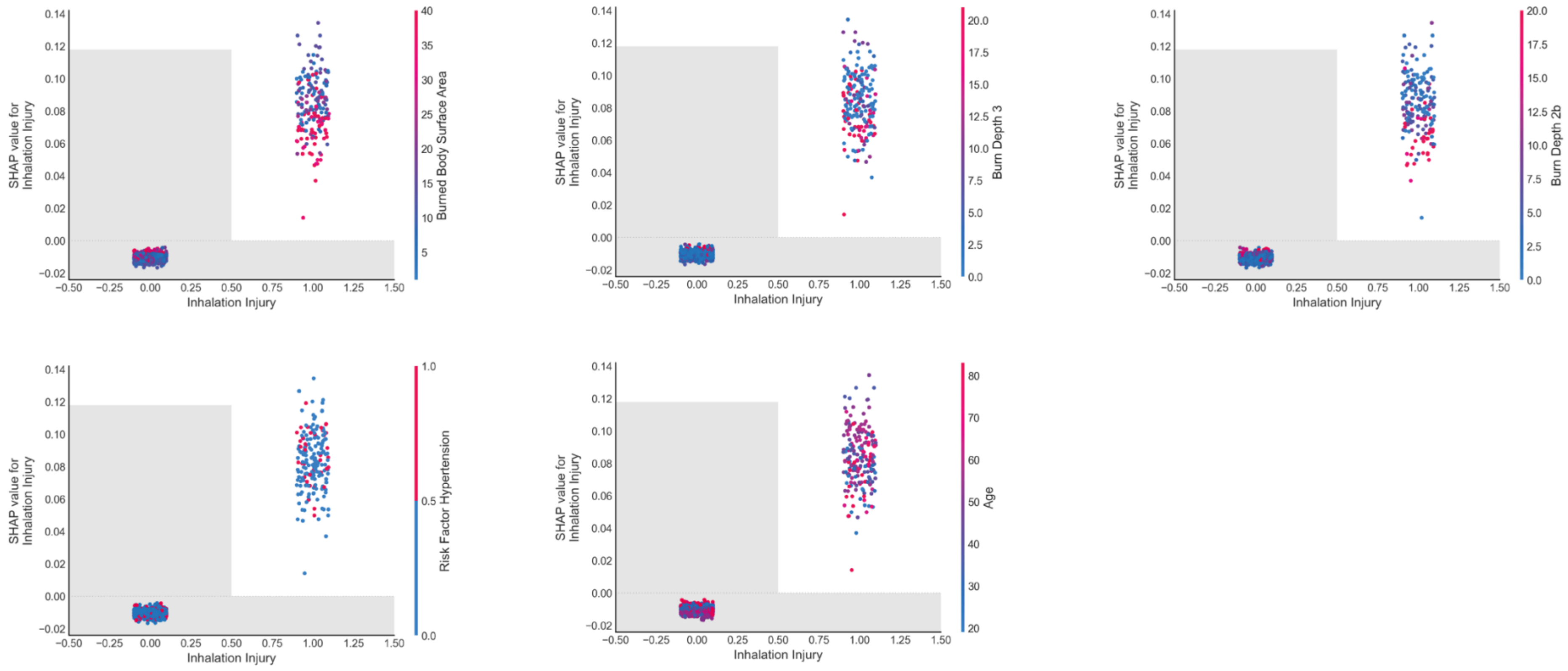

Supplementary Figure 2. SHAP Dependence Plots for Inhalation Injury. SHAP dependence plots illustrating the interaction of Inhalation Injury with another model feature, where the dot color corresponds to the value of that feature (Burned Body Surface Area, Burn Depth 3, Burn Depth 2b, Hypertension, and Age). The SHAP value of Inhalation Injury is shown on the x-axis (0 = no Inhalation Injury, 1 = Inhalation Injury present), and its impact on the prediction is shown on the y-axis (SHAP value).

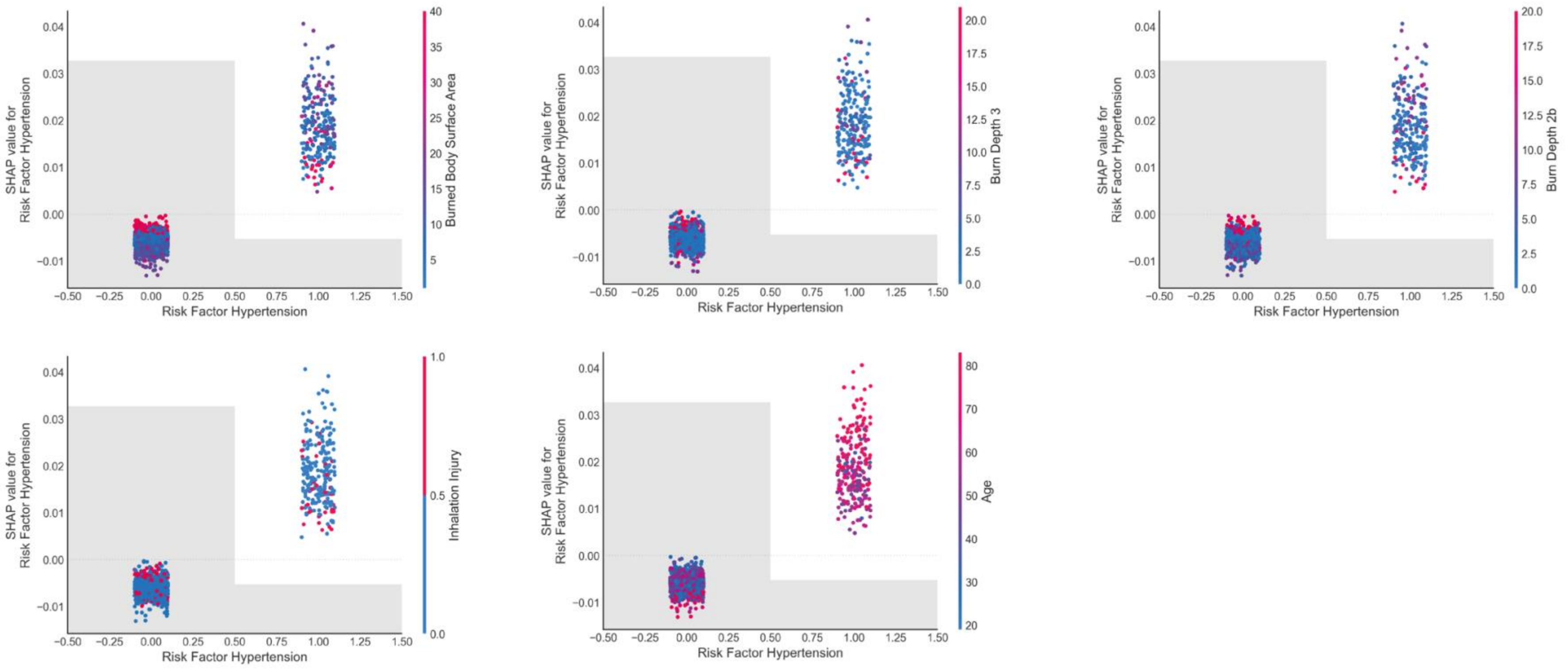

Supplementary Figure 3. SHAP Dependence Plots for Hypertension. SHAP dependence plots illustrating the interaction of Inhalation Injury with another model feature, where the dot color corresponds to the value of that feature (Burned Body Surface Area, Burn Depth 3, Burn Depth 2b, Inhalation Injury, and Age). The SHAP value of Hypertension is shown on the x-axis (0 = no Hypertension, 1 = Hypertension present), and its impact on the prediction is shown on the y-axis (SHAP value).

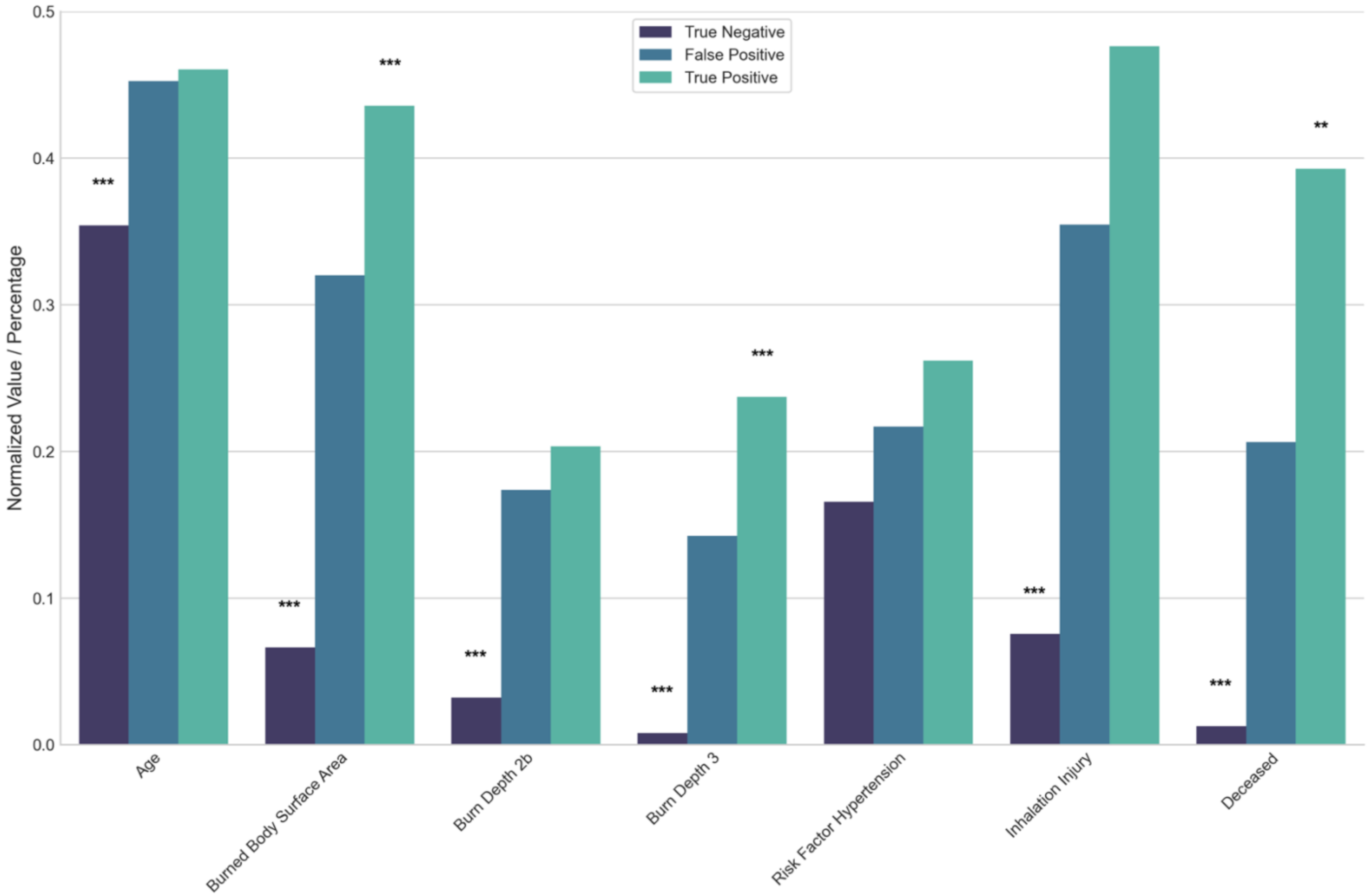

Supplementary Figure 4. Normalized Comparison of Key Characteristics Across Prediction Groups (Random Forest with EDA Dataset). Bar chart comparing the mean values of continuous variables (normalized to a 0-1 scale) and the percentage of categorical variables across the True Negative (TN), False Positive (FP), and True Positive (TP) groups. The FP group consistently represents an intermediate profile between the TN and TP groups. Significance markers (*p*<0.05, **p*<0.01, ***p*<0.001) indicate a statistically significant difference compared to the False Positive (FP) group.

Supplementary Review: Current Standard of Care for Burned Patients in Germany

The management of severely burned patients in Germany is a highly specialized and structured discipline. While primarily guided by the national S2k-level guideline from the German Society for Burn Medicine (DGV)[1], its core principles are reflected in broader European guidelines and the international literature. This framework ensures a standardized, evidence-based approach, though the field is dynamic, and individual expert centers may adopt evolving techniques based on local expertise and emerging evidence.

Fluid Resuscitation

The initial management of burn shock follows a strategy of goal-directed fluid therapy rather than rigid adherence to formulas. While formulas such as Parkland-Baxter or modified Brooke are used to calculate an initial infusion rate, the standard of care is immediate and continuous titration of fluids to maintain specific physiological endpoints, most commonly a urine output of 0.5–1.0 ml/kg/hour[1]. In complex cases, advanced hemodynamic monitoring (e.g., transpulmonary thermodilution) may also be used to guide therapy[2,3].

The fluid of choice for initial resuscitation (the first 8 hours) is a balanced, plasma-adapted crystalloid solution (e.g., Ringer's acetate). After the first 8–24 hours, the introduction of human albumin (5% or 20%) is recommended for patients with persistent hemodynamic instability or crystalloid requirements significantly exceeding initial estimates. This strategy aims to restore plasma oncotic pressure and mitigate the effects of "fluid creep" (over-resuscitation) and associated complications like compartment syndromes and pulmonary edema. Artificial colloids such as hydroxyethyl starch (HES) are contraindicated[1,4].

Wound Management and Therapeutic Strategies

A distinction is made based on burn depth. Superficial partial-thickness (Grade 2a) burns generally do not pose a significant problem and are treated conservatively, often with antiseptic dressings or, depending on the center, specific wound coverings like Suprathel®[5]. In contrast, the guiding principle for deep partial-thickness (Grade 2b) and full-thickness (Grade 3) burns is early excision and wound closure. The goal is to perform the first debridement within 24–72 hours post-injury, contingent on patient stabilization, to remove the necrotic eschar which serves as a nidus for infection and systemic inflammation[6]. However, clinical complexities often lead to delays, with fewer than 50% of patients undergoing excision within this ideal window[7].

Two main debridement strategies are employed for deeper burns. The primary surgical method is traditional tangential excision, where necrotic tissue is sequentially shaved away. For deeper injuries, epifascial excision down to the muscle fascia is sometimes performed, though this has

become less frequent[6]. Increasingly, as an alternative to surgery, centers may use selective enzymatic debridement with a bromelain-based agent (NexoBrid®). This technique digests necrotic eschar while preserving viable tissue, significantly reducing the need for surgery and autografting, minimizing blood loss, and proving particularly useful for burns in functionally and cosmetically sensitive areas like the hands and face[8].

Following debridement, a spectrum of wound closure techniques is applied. For some deep partial-thickness (Grade 2b) wounds, particularly after enzymatic debridement, a trial of conservative management with advanced temporary skin substitutes (e.g., Suprathel®[5], Kerecis® Omega3 Wound[9]) may be initiated to promote spontaneous healing. When conservative treatment fails or is not indicated, definitive wound closure is achieved with a split-thickness skin graft (STSG)[10]. In full-thickness injuries (Grade 3), a staged reconstruction is often necessary. This involves the initial application of a dermal substitute (e.g., Matriderm®[11], Integra®[12], BTM®[13]) to regenerate a neodermis, which is then covered with a subsequent STSG to provide the final epidermal layer[14].

Common Sources of Sepsis and Causative Organisms

Infectious complications, primarily sepsis, are the leading cause of late mortality in German burn ICUs[15,16]. The diagnosis is challenging due to the systemic inflammatory response syndrome (SIRS) caused by the burn itself, which mimics clinical signs of infection. The three most common loci of infection are the burn wound itself, where colonization of the avascular eschar can progress to invasive infection; the lungs, leading to ventilator-associated pneumonia (VAP) from prolonged mechanical ventilation; and the bloodstream, via catheter-related bloodstream infections (CLABSI) from the requisite use of central venous catheters for resuscitation and support[17].

The microbiology of burn-related infections follows a predictable temporal pattern. The initial phase (first 3–5 days) is dominated by Gram-positive organisms, primarily *Staphylococcus aureus*, originating from the patient's endogenous skin flora[17,18]. After this period, the landscape shifts to predominantly Gram-negative bacteria, which are often multidrug-resistant (MDR) nosocomial pathogens. The most common isolates include *Pseudomonas aeruginosa*, *Acinetobacter baumannii*, and *Klebsiella pneumoniae*. Fungal infections, typically with *Candida* species, are a concern in the late phase of care, especially after prolonged broad-spectrum antibiotic use[15,17,18].

References

1. German Society of Burn Medicine. Treatment of thermal injuries in adults. AWMF Guideline No. 044-001 (S2k). Published February 2021. Available from: https://register.awmf.org/de/leitlinien/detail/044-001

2. Kraft, R. *et al.* OPTIMIZED FLUID MANAGEMENT IMPROVES OUTCOMES OF PEDIATRIC BURN PATIENTS. (2012) doi:10.1016/j.jss.2012.05.058.

3. Gwyn-Jones, A. *et al.* Major burns in adults: a practice review. *Emergency Medicine Journal* **41**, 630–634 (2024).

4. European Burns Association European Practice Guidelines for Burn Care Minimum level of Burn Care Provision in Europe. (2017).

5. Keck, M. *et al.* The use of Suprathel(®) in deep dermal burns: first results of a prospective study. *Burns* **38**, 388–395 (2012).

6. Jeschke, M. G. *et al.* Burn injury. *Nat Rev Dis Primers* **6**, (2020).

7. Ziegler, B. *et al.* Plastic Surgery. **28**, 232–242 (2020).

8. Hirche, C. *et al.* Eschar removal by bromelain based enzymatic debridement (Nexobrid 1) in burns: European consensus guidelines update. (2020) doi:10.1016/j.burns.2020.03.002.

9. Wallner, C. *et al.* The Use of Intact Fish Skin as a Novel Treatment Method for Deep Dermal Burns Following Enzymatic Debridement: A Retrospective Case-Control Study. *European Burn Journal 2022, Vol. 3, Pages 43-55* **3**, 43–55 (2022).

10. Rowan, M. P. *et al.* Burn wound healing and treatment: review and advancements. *Crit Care* **19**, (2015).

11. Haslik, W. *et al.* First experiences with the collagen-elastin matrix Matriderm as a dermal substitute in severe burn injuries of the hand. *Burns* **33**, 364–368 (2007).

12. Heimbach, D. M. *et al.* Multicenter postapproval clinical trial of Integra dermal regeneration template for burn treatment. *J Burn Care Rehabil* **24**, 42–48 (2003).

13. Struble, S. L. *et al.* Outcomes of Biodegradable Temporizing Matrix for Soft Tissue Reconstruction of the Hand and Extremities. *Plast Reconstr Surg Glob Open* **12**, e5956 (2024).

14. Brusselaers, N. *et al.* Skin replacement in burn wounds. *J Trauma* **68**, 490–501 (2010).

15. Boehm, D. & Menke, H. Sepsis in Burns—Lessons Learnt from Developments in the Management of Septic Shock. *Medicina (B Aires)* **58**, 26 (2021).

16. Bloemsma, G. C., Dokter, J., Boxma, H. & Oen, I. M. M. H. Mortality and causes of death in a burn centre. *Burns* **34**, 1103–1107 (2008).

17. Church, D., Elsayed, S., Reid, O., Winston, B. & Lindsay, R. Burn wound infections. *Clin Microbiol Rev* **19**, 403–434 (2006).

18. Slaviero, L., Avruscio, G., Vindigni, V. & Tocco-Tussardi, I. Antiseptics for burns: a review of the evidence. *Ann Burns Fire Disasters* **31**, 198 (2018).
